# Purification and characterization of a novel anti-coagulant from the leech Hirudinaria manillensis

**DOI:** 10.24272/j.issn.2095-8137.2019.037

**Published:** 2019-05-18

**Authors:** Ruo-Mei Cheng, Xiao-Peng Tang, Ai-Lin Long, James Mwangi, Ren Lai, Rui-Pu Sun, Cheng-Bo Long, Zhen-Qing Zhang

**Affiliations:** 1 Department of Pharmaceutical Sciences, College of Pharmaceutical Sciences, Soochow University, Suzhou Jiangsu 215123, China; 2 Key Laboratory of Animal Models and Human Disease Mechanisms of Chinese Academy of Sciences/Key Laboratory of Bioactive Peptides of Yunnan Province, Kunming Institute of Zoology, Kunming Yunnan 650223, China; 3 Kunming College of Life Science, University of Chinese Academy of Sciences, Kunming Yunnan 650204, China; 4 Jiangxi Medical College, Nanchang University, Nanchang Jiangxi 330006, China

**Keywords:** *Hirudinaria manillensis*, Bdellin-HM-2, “Non-classical” Kazal inhibitors, Blood sucking, Anticoagulant, Anti-thrombotic drugs

## Abstract

Protease inhibitors have been reported rarely from the leech *Hirudinaria manillensis*. In this study, we purified a novel protease inhibitor (bdellin-HM-2) with anticoagulant properties from *H. manillensis*. With a molecular weight of 1.4x10^4^, bdellin-HM-2 was also characterized with three intra-molecular disulfide bridges at the N-terminus and multiple HHXDD and HXDD motifs at the C-terminus. cDNA cloning revealed that the putative nucleotide-encoding protein of bdellin-HM-2 contained 132 amino acids and was encoded by a 399 bp open reading frame (ORF). Sequence alignment showed that bdellin-HM-2 shared similarity with the “non-classical” Kazal-type serine protease inhibitors, but had no inhibitory effect on trypsin, elastase, chymotrypsin, kallikrein, factor XIIa (FXIIa), factor XIa (FXIa), factor Xa (FXa), thrombin, or plasmin. Bdellin-HM-2 showed anticoagulant effects by prolonging the activated partial thromboplastin time (aPTT), indicating a role in enabling *H. manillensis* to obtain a blood meal from its host. Our results suggest that bdellin-HM-2 may play a crucial role in blood-sucking in this leech species and may be a potential candidate for the development of clinical anti-thrombotic drugs.

## INTRODUCTION

Protease inhibitors occur naturally in living organisms, including animals (Shadrin et al., 2015; Vicuna et al., 2015; Wang et al., 2005; Zhang, 2006), plants (Kim et al., 2009; Ryan, 1990), fungi (Sabotic & Kos, 2012), and bacteria (Supuran et al., 2002). They have multifunctional roles in many physiological processes and play an important role in biological functions of venomous animals, such as in predation (Birrell et al., 2007) and defense (Ali et al., 2002). To prevent clotting during blood feeding from a host, hematophagous animals have developed various mechanisms to interfere with blood coagulation (Markwardt, 1996). Among the inhibitors involved in coagulation, protease inhibitors are the most prominent anticoagulants currently described and characterized from leeches (Dodt, 1995) and insects (De Marco et al., 2010; Mende et al., 1999).

There are at least four types of protease inhibitors, including serine, cysteine, aspartic, and metalloprotease inhibitors (Leung et al., 2000). The Kazal family is one of the best-known groups of serine protease inhibitors (Rimphanitchayakit & Tassanakajon, 2010). Kazal-type inhibitory can be sorted into classical and non-classical Kazal domains. The classical Kazal domain has two residues between cys4 and cys5, whereas the non-classical Kazal inhibitor has a spacer region between cys4 and cys5, ranging from three to seven residues (Moser et al., 1998). There are highly homologous three-dimensional structures in the Kazal-type serine proteinase inhibitors regardless of length of amino acid sequences between the cysteines and amino acid sequence variation (Eigenbrot et al., 2012). The P1 residue, located in the second amino acid downstream of the second conserved cysteine residue, is inserted into the S1 specificity pocket of the protease in a substrate-like way (Bode & Huber, 1992; Laskowski & Kato, 1980).

Several Kazal-type serine protease inhibitors have been characterized from leeches. A few “non-classical” Kazal inhibitors have been reported from different leeches, including bdellin-B-3 (Fink et al., 1986), bdellin-KL (Kim et al., 2001), and bdellin-HM (Lai et al., 2016). In this study, bdellin-HM-2 was purified and characterized from the leech *H. manillensis*. To the best of our knowledge, bdellin-HM-2 is the first Kazal-type serine protease inhibitor displaying anticoagulant properties identified from *H. manillensis*.

## MATERIALS AND METHODS

### Collection of crude extracts

The *H. manillensis* leeches were purchased from Jinbian aquafarm, Qinzhou City, Guangxi Province in China. The leeches were still alive when transported to the laboratory. We prepared the crude extracts from the leech heads as described previously (Lai et al., 2016). In short, leech heads were separated from the bodies, washed in 0.9% saline, quickly frozen, and then ground in liquid nitrogen.

### Purification of bdellin-HM-2

Purification of bdellin-HM2 followed similar methods described in our previous published article (Lai et al., 2016). Briefly, crude extracts were dissolved in 50 mmol/L Tris-HCl buffer (pH 8.9) and subsequently separated by a DEAE Sephadex A-50 column (5 cm diameter, 60 cm length, GE, USA). Elution was performed at a flow rate of 15 mL/h at 4 °C and 3.0 mL fractions were collected in separate tubes. The absorbance of the fractions was monitored at both 215 and 280 nm. Fractions that could prolong the activated partial thromboplastin time (aPTT) were pooled and lyophilized prior to further purification. The powder from the previous step was dissolved and loaded for reverse-phase high-performance liquid chromatography (RP-HPLC) on a C_18_ column (Waters, Milford, MA, USA, 5 μm particle size, 250 mm×4.6 mm). Elution was carried out with a linear gradient of 10%–60% solution B (99.9% acetonitrile, 0.1% TFA) for 60 min at a flow rate of 1 mL/min. The eluted fraction that prolonged aPTT was collected.

### Mass spectrometric analysis and peptide sequencing

The molecular weight of the collected fraction was analyzed by matrix-assisted laser desorption ionization time-of-flight mass spectrometry (MALDI-TOF-MS, Autoflex speed TOF/TOF, Bruker Daltonik GmbH, Bruker Corporation, Germany) using positive ion and linear mode, with specific operating parameters including a 20 kV ion acceleration voltage, 50-time accumulation for single scanning, and 0.1% accuracy of mass determinations. The partial peptide sequence of the N-terminal was determined by automatic Edman degradation on a pulsed liquid-phase sequencer (PPSQ-31A, Shimadzu Corporation, Japan).

### RNA extraction and cDNA library construction

Total RNA from the head of *H. manillensis* was extracted using Trizol reagent (Life Technologies, Carlsbad, CA, USA) according to the manufacturer’s instructions and was dissolved in RNase-Free water. A SMART™ PCR cDNA construction kit (Clontech, Palo Alto, CA, USA) was used for synthesizing cDNA, as described previously (Lai et al., 2016).

### Screening of cDNA encoding bdellin-HM-2

To screen the cDNA encoding the precursor of bdellin-HM-2, synthesized cDNA was used as the template for PCR, following previously described methods (Lai et al., 2016). Briefly, two pairs of oligonucleotide primers (Table 1) were used in PCR reactions, where primers 1 and 3 were designed according to the partial N-terminal sequence of bdellin-HM-2 and primers 2 and 4 were from the SMART™ PCR cDNA construction kit. The PCR conditions were as described previously (Lai et al., 2016).

**Table 1:** Primers used for cDNA cloning of bdellin-HM-2

Primer	Sequence (5'–3')
1	AACAGGTTTGCGGAAGT
2	AAGCAGTGGTATCAACGCAGAGT
3	AATTCCAGGGTACAGACG
4	ATTCTAGAGGCCGAGGCGGCCGA

Primers 1 and 2 for signal peptide cloning; primers 3 and 4 for mature peptide cloning.

### Effects of bdellin-HM-2 on blood coagulation

For aPTT assay, the aPTT reagent (50 μL, F008-1, Nanjing Jiancheng Bioengineering Institute, China) was incubated with 50 μL of plasma and different concentrations of bdellin-HM-2 (0.7 and 1.4 μmol/L). After 3-min incubation, CaCl_2_ (50 μL, 25 mmol/L) preheated at 37 °C for 5 min was added, and the clotting curve was monitored at 650 nm using an enzyme-labeled instrument (Epoch BioTek, USA) for 2 min. To test the prothrombin time (PT), plasma (50 μL) was incubated with different concentrations of bdellin-HM-2 (0.7 and 1.4 μmol/L) for 3 min at 37 °C, after which the PT reagent (100 μL, F007, Nanjing Jiancheng Bioengineering Institute, China) preheated at 37 °C for 15 min was added and the clotting curve was monitored at 650 nm using the enzyme-labeled instrument for 30 s.

### Effects of bdellin-HM-2 on proteases

Effects of bdellin-HM-2 on proteases, including trypsin, elastase, chymotrypsin, kallikrein, factor XIIa (FXIIa), factor XIa (FXIa), factor Xa (FXa), thrombin, and plasmin were tested using the corresponding chromogenic substrates. The testing enzyme was incubated with different concentrations (0, 0.7, 1.4, 2.8, and 5.6 μmol/L) of bdellin-HM-2 in 60 μL of 50 mmol/L Tris buffer (pH 7.4) for 5 min, with a certain concentration of chromogenic substrate then added. Absorbance at 405 nm was monitored immediately and the kinetic curve was recorded using the enzyme-labeled instrument for 30 min. Bovine pancreas trypsin, elastase, chymotrypsin, and plasmin were all obtained from Sigma (USA) and the enzyme concentrations used were 800, 400, 400, and 20 nmol/L, respectively. The corresponding chromogenic substrates (Sigma, USA) were Gly-Arg-*p*-nitroanilide dihydrochloride for trypsin, *N*-methoxysuccinyl-Ala-Ala-Pro-Val-*p*-nitroanilide for elastase, *N*-succinyl-Gly-Gly-Phe-*p*-nitroanilide for chymotrypsin, and Gly-Arg-*p*-nitroanilide dihydrochloride for plasmin. The concentration of all substrates in the reactions was 0.2 mmol/L. The concentrations used for kallikrein, FXIa, and FXa (Enzyme Research Laboratory, USA) were 400, 400, and 20 nmol/L, respectively, and the corresponding chromogenic substrates were *H-D*-Pro-Phe-Arg-*p*NA·2HCl (Hyphen Biomed, France), *H-D*-Pro-Phe-Arg-*p*NA·2HCl (Hyphen Biomed, France), and CH_3_OCO-*D*-CHA-Gly-Arg-*p*NA-AcOH (Sigma, USA), respectively. The concentration of all three substrates in the reaction was 0.2 mmol/L. Human thrombin (Sigma, USA, 10 nmol/L) and FXIIa (Enzyme Research Laboratories, USA, 10 nmol/L) were reacted with 0.2 mmol/L chromogenic substrate of *H-D*-Phe-Pip-Arg-*p*Na·2HCl (Hyphen Biomed, France) and *H-D*-Pro-Phe-Arg-*p*NA·2HCl (Hyphen Biomed, France), respectively.

## RESULTS

### Purification of bdellin-HM-2

**Figure 1:**
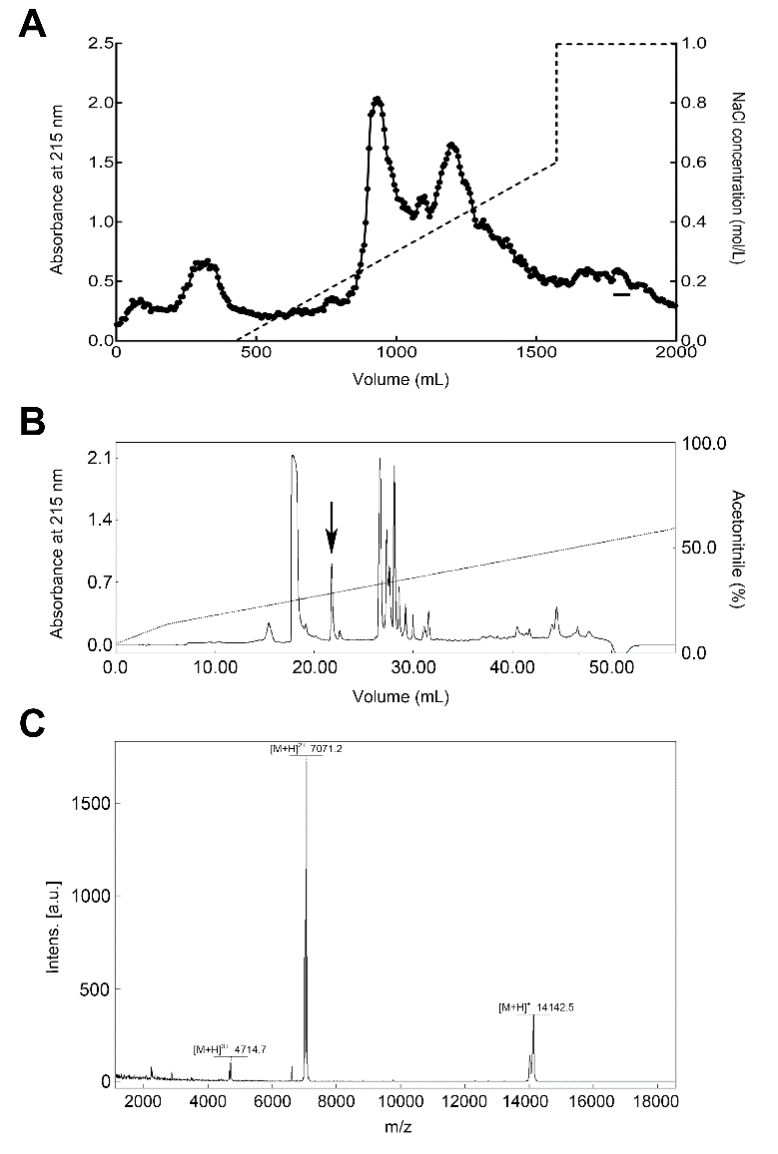
Purification of bdellin-HM-2 from *H. manillensis*

The crude extracts from *H. manillensis* were resolved into several fractions by DEAE Sephadex A-50 column. The fraction that prolonged the aPTT was indicated by a bar (Figure 1A). We then obtained the purified peptide exerting an aPTT inhibitory effect, named bdellin-HM-2 (indicated by an arrow in Figure 1B), using a C_18_ RP-HPLC column. MALDI-TOF-MS showed that bdellin-HM-2 had a molecular weight (MW) of 14141.5 (Figure 1C).

A: Crude extracts were fractionated using DEAE Sephadex A-50 ion exchange. Fraction exerting inhibitory activity on aPTT is indicated by a bar (–). B: Fraction exerting inhibitory activity on aPTT was further purified by C_18_ reverse-phase high-performance liquid chromatography (RP-HPLC). Protein peak exerting inhibitory activity on the aPTT is marked by an arrow. C: Matrix-assisted laser desorption ionization time-of-flight (MALDI-TOF) analysis of purified native bdellin-HM-2.

### Primary structure of bdellin-HM-2

**Figure 2:**
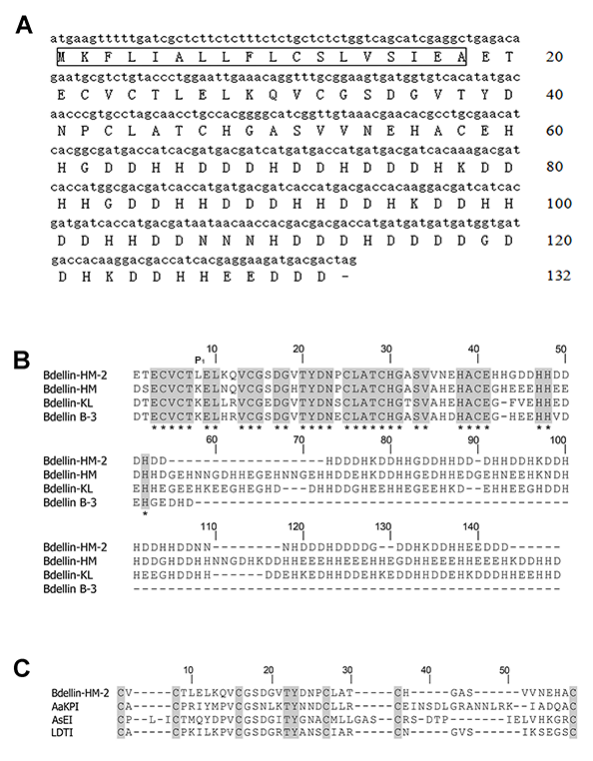
cDNA sequence encoding bdellin-HM-2 precursor and sequence alignment with other protease inhibitors

Based on automatic Edman degradation, the partial N-terminal sequence of bdellin-HM-2 was determined to be ETECVCTLELKQVCGS. According to the N-terminal sequence, degenerate primers were designed (Table 1) to clone the cDNA encoding the precursor of bdellin-HM-2 from the cDNA library. A 399 bp cDNA encoding the precursor of bdellin-HM-2 was obtained. The cDNA had an open reading frame (ORF) of 396 nucleotides coding a pro-protein of 132 amino acids, including a signal peptide of 18 residues (indicated by box) and mature bdellin-HM-2 of 114 residues (Figure 2A). The theoretical MW of mature bdellin-HM-2 was 13144.78, which was not consistent with the observed mass by mass spectrometry analysis (Figure 1C). This inconsistency may be due to post-translational modification of the protein. Sequence alignment showed similarity to bdellin-KL (Kim et al., 2001), bdellin-B-3 (Fink et al., 1986), and bdellin-HM (Lai et al., 2016), which are “non-classical” Kazal serine protease inhibitors (Figure 2B). Multiple sequence alignment showed that the six cysteine residues and threonine-tyrosine residues were highly conserved among different species (Figure 2C). There were multiple HHXDD and HXDD motifs at the C-terminus of bdellin-HM-2.

A: Nucleotide sequence encoding bdellin-HM-2 precursor and deduced amino acid sequence. Signal peptide is boxed. Bar (–) indicates stop codon. B: Sequence comparison of bdellin-HM-2 with bdellin-HM, bdellin-KL, and bdellin-B-3. Identical amino acid residues are indicated by an asterisk (*). C: Multiple sequence alignment of Kazal domain from *H. manillensis* (bdellin-HM-2), *Aedes aegypti* (AaKPI ABF18209), *Anemonia sulcata* (AsEI 1Y1B), and *H. medicinalis* (LDTI P80424). Conserved threonine-tyrosine residues between cysteine 3 and 4 are indicated. Conserved cysteine motifs are also indicated.

### Anticoagulant activity of bdellin-HM-2

**Figure 3:**
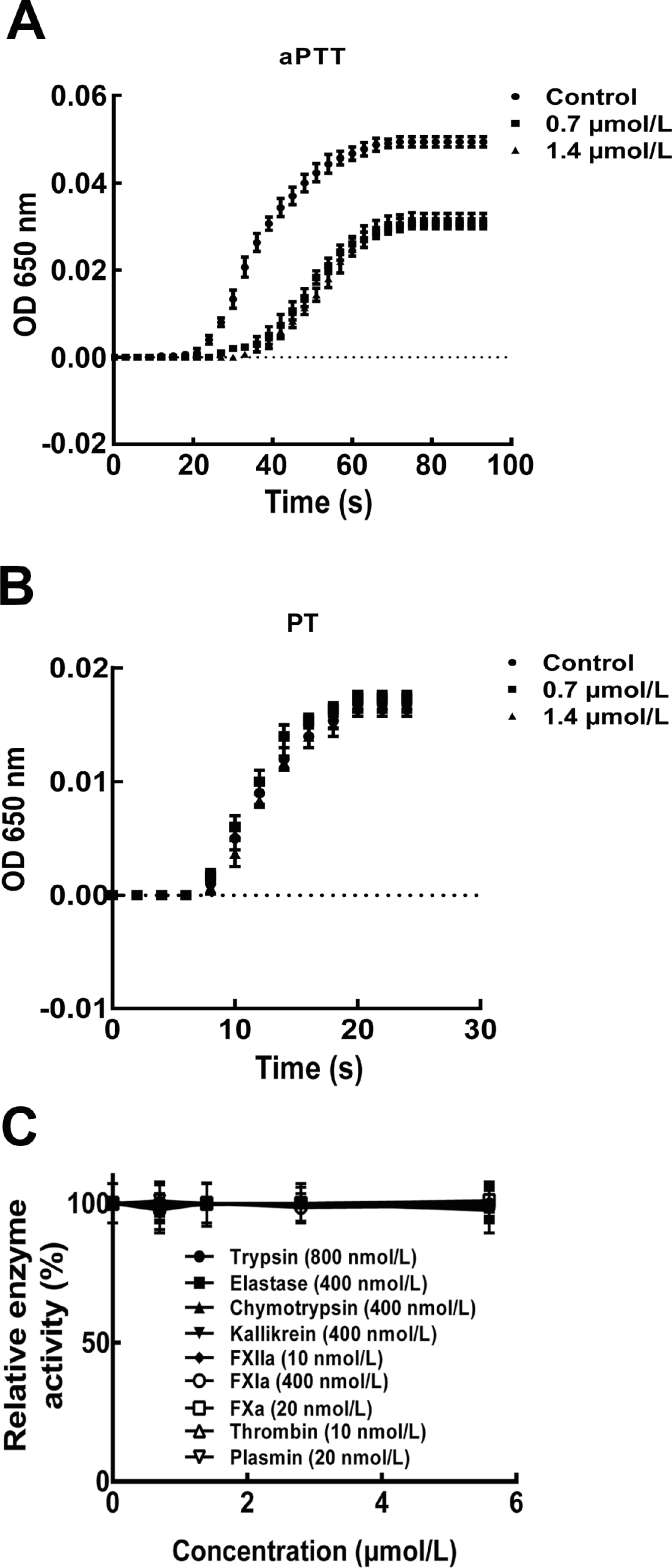
Effects of bdellin-HM-2 on aPTT

**Figure 4:**
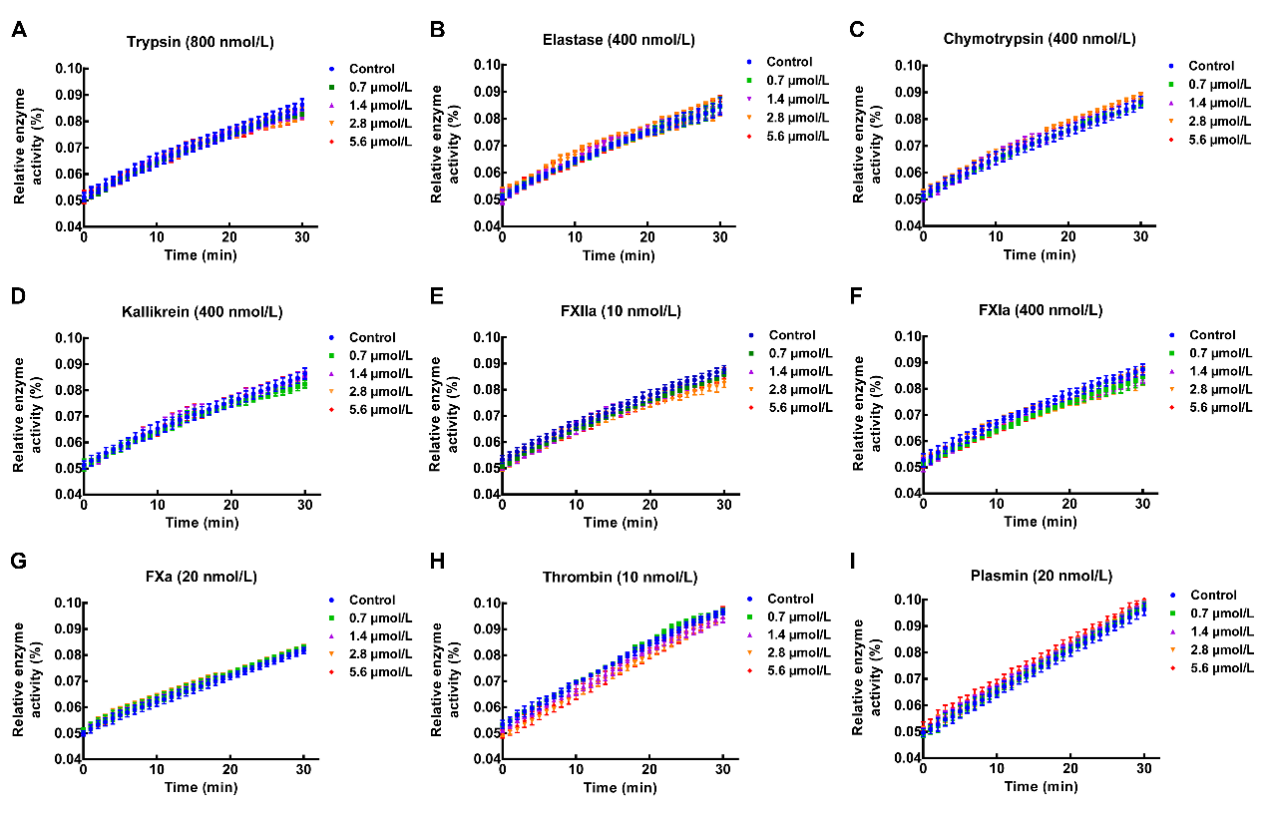
Bdellin-HM-2 had no effect on proteases

Under the assay conditions, bdellin-HM-2 exerted anticoagulatory activity by inhibiting aPTT (Figure 3A), whereas no inhibitory activity was observed on PT (Figure 3B). Compared with the control with an aPTT of ~60 s, the aPTT was prolonged to ~100 s after 0.7 and 1.4 μmol/L bdellin-HM-2 treatment, suggesting that bdellin-HM-2 acts on the intrinsic pathway. Bdellin-HM-2 had no effect on trypsin, elastase, chymotrypsin, kallikrein, FXIIa, FXIa, FXa, thrombin, or plasmin (Figure 3C). All enzyme activity test results were plotted (Figure 4).

Bdellin-HM-2 action on aPTT (A) and PT (B). C: Bdellin-HM-2 effects on trypsin, elastase, chymotrypsin, kallikrein, FXIIa, FXIa, FXa, thrombin, and plasmin were analyzed by hydrolysis of chromogenic substrates. Data are means±*SD* of six independent experiments.

Bdellin-HM-2 effects on trypsin (A), elastase (B), chymotrypsin (C), kallikrein (D), FXIIa (E), FXIa (F), FXa (G), thrombin (H), and plasmin (I). Data represent at least six independent experiments and are presented as means±*SD*.

## DISCUSSION

Several protease inhibitors exerting anticoagulant effects have been found from leeches (Hong & Kang, 1999; Markwardt, 2002; Salzet et al., 2000; Salzet, 2001; Strube et al., 1993; Tuszynski et al., 1987). In this report, a novel protease inhibitor (bdellin-HM-2) with anticoagulant effects was purified and further characterized from *H. manillensis* for the first time. The cDNA encoding bdellin-HM-2 precursor was cloned from the cDNA library, and the mature bdellin-HM-2 consisted of 114 amino acid residues. MALDI-TOF-MS showed that the MW of bdellin-HM-2 was 14141.5, compared to the theoretical molecular weight of 13144.78, a difference of 996.72, which is not consistent with the theoretical value. Research shows glycosylation influences the function of protein, governs physiology, and contributes to disease (Ohtsubo & Marth, 2006). We speculated that bdellin-HM-2 was O-glycosylated at Thr-20, Thr-25, Ser-34, Thr-38, and Thr-46. (Gupta & Brunak, 2002), although further research on these O-glycosylation sites should be performed in the future.

Kazal-type inhibitors with one or more Kazal domains are characterized by multiple HHXDD and HXDD motifs in their amino acid sequences (Laskowski & Kato, 1980) and by their highly homologous three-dimensional structures (Van et al., 1995). Each Kazal domain usually contains six conserved cysteine residues forming three intra-molecular disulfide bridges (Laskowski & Kato, 1980; Magert et al., 1999). P1 residue, which contributes to the inhibitory specificity, is located at the second position after the second cysteine residue of the Kazal domain (Bode & Huber, 1992). Although bdellin-HM-2 showed high similarity to bdellin-HM and bdellin-KL by sequence analysis and belongs to the family of non-classical Kazal domains, enzyme activity tests showed that bdellin-HM-2 had no inhibitory effects on trypsin, elastase, chymotrypsin, kallikrein, FXIIa, FXIa, FXa, thrombin, or plasmin under the assay conditions. Sequence alignment showed that the P1 residue of bdellin-HM-2 was different from bdellin-HM, bdellin-KL, and bdellin-B-3. The difference in P1 residue was considered the cause of the enzyme activity test results.

Bdellin-HM-2 prolonged the aPTT, implying that bdellin-HM-2 functioned to help *H. manillensis* obtain a blood meal by inhibiting blood coagulation. Results showed that the activity was dose-independent. Further work to identify the target of bdellin-HM-2 in blood is necessary. Blood-sucking animals obtain a blood meal by overcoming the host’s natural blood coagulation (De Marco et al., 2010; Dodt, 1995; Markwardt, 1996; Mende et al., 1999). The anticoagulant peptide obtained from *H. manillensis* not only facilitates our understanding of the mechanism of blood feeding for *H. manillensis*, but also provides a new candidate for the development of clinical anticoagulant drugs.

In conclusion, bdellin-HM-2 identified from *H. manillensis* prolonged the aPTT but exhibited no influence on PT and no inhibitory activity on trypsin, elastase, chymotrypsin, kallikrein, FXIIa, FXIa, FXa, thrombin, or plasmin under the assay conditions. Further research on O-glycosylation sites will be performed in the future. Bdellin-HM-2 is the first identified Kazal-type serine protease inhibitor from *H. manillensis* that shows a potent anticoagulant effect.
